# Supplementary material for: SyNDI: synchronous network data integration framework
Source: BMC Bioinformatics. 2018 Nov 6;19:403. doi: 10.1186/s12859-018-2426-5 (PMC6219086; doi:10.1186/s12859-018-2426-5)
Supplement: Supplementary file 5 — Files of the example presented in Additional file 4. This additional file is a zip package that contains all files used in Additional file 4. Detailed descriptions of each of these files are presented in a README file included in this zip package. (GZ 30411 kb) [file 12859_2018_2426_MOESM5_ESM.gz › SyNDITools/Meme2Fimo/meme_4.9.0_4.tar.gz!/meme_4.9.0/website/html/meme-faq.html]

MEME - FAQ


|  |  |
| --- | --- |
|  | MEME Suite The MEME Suite web application requires the use of JavaScript   Javascript doesn't seem to be available on your browser.  Frequently Asked Questions     ---    - **Q.** Why don't the motif occurrences in   the "Summary of Motifs" block diagrams match the occurrences shown   in the individual motif block diagrams?  ---     - **Q.** Why don't the motif occurrences in the "Summary of   Motifs" block diagrams match the occurrences shown in the   individual motif block diagrams? **A.** This is a feature, not a bug. The motif occurrences   shown for a motif in its section of the output are the ones   discovered by the MEME algorithm. These occurrences are then used   to create a position specific scoring matrix (PSSM) for the motif.   The PSSMs for all of the motifs discovered by MEME are then used to   search for non-overlapping sets of occurrences of motifs with   p-values under 0.0001 using the MAST algorithm. These are displayed   in the "Summary of Motifs" block diagram. Individual motif   occurrences will sometimes be missing from the "Summary of Motifs"   block diagram because of the non-overlapping or minimum p-value   requirements. Conversely, there may be motif occurrences in the   summary diagram that were not discovered by the MEME algorithm.  ---  Click here for **more information** on **MAST**.  Return to **MEME Suite introduction**. |
